# Ocular Vascular Changes: Choroidal Thickness as an Early Biomarker for Alzheimer’s Disease?

**DOI:** 10.3390/jpm11121365

**Published:** 2021-12-14

**Authors:** Chiara Villa

**Affiliations:** School of Medicine and Surgery, University of Milano-Bicocca, 20900 Monza, Italy; chiara.villa@unimib.it

Alzheimer’s disease (AD) is an age-related neurodegenerative and progressive disorder representing the most common form of dementia among the elderly [1]. As the global population ages, the number of people suffering from AD rises rapidly, making this disorder a great public health issue with significant economic burden [2]. AD is clinically characterized by memory loss and other cognitive functions, behavioural changes and significant cognitive impairment, with associated neuropsychological manifestations [3]. Although behavioural symptoms can be alleviated by the current therapeutic strategies, no drug is available to prevent or halt the disease progression [4]. The established biomarkers in daily clinical practice provide early identification of the two neuropathological hallmarks of AD, the β-amyloid (Aβ) plaques and the neurofibrillary tangles (NFTs) composed of filamentous hyperphosphorylated tau proteins, detected by cerebrospinal fluid (CSF) examinations and advanced neuroimaging techniques like positron magnetic resonance imaging (MRI) and positron emission tomography (PET). However, these evaluations are invasive, expensive and laborious, restricting their wide use at the early stages of the disease [5]. Over recent years, ocular biomarkers have received great interest as they can be visualized by non-invasive, rapid and cost-efficient imaging platforms, such as optical coherence tomography (OCT) and fundus camera imaging [6].

AD-related pathology is often accompanied by vascular Aβ deposition, known as cerebral amyloid angiopathy (CAA) [7]. The presence of Aβ aggregates can induce vascular changes by replacing the contractile smooth musculature of the arteriole as well as the small and medium calibre artery walls [7,8,9]. These alterations appear not only in the brain vascular system, but also in the eye, impairing the regulation of the local blood flow and decreasing the vascular density and thickness [10]. Interestingly, the brain and eye, in particular the retina, have some similarities: the same embryonic origin and the complex composition of neuronal tissue and glial cells [11]. Regarding AD, increasing evidences reported that the neuronal and retinal microvasculature share similar neurobiology, so modifications of the retina may mirror pathological processes occurring in the development of the disorder. In patients with mild cognitive impairment (MCI), a thickness of the retina and choroid, a decreased vessel complexity and a reduced blood flow have been shown, suggesting their potentiality as early biomarkers for AD [12]. However, it should be taken in consideration that two different vascular systems are responsible for ocular irrigation: the central retinal artery (CRA) and the ciliary arteries. The inner retinal layers are supplied by their own blood derived from the CRA with a high ratio of endothelial cell/pericyte while the outer retinal layers are supported only by the capillaries of the choroid with an endothelial cell/pericyte ratio of 6:1. Therefore, these two ocular vascular systems differ in the regulation of blood flow and perfusion pressure, so they could react differently to a pathological event [13]. Also, alterations in optic nerve have been related to cognitive decline and brain abnormalities observed in early AD [14]. The perfusion of optic nerve is conditioned not only by the blood flow and oxygen saturation but also by haemoglobin (Hb) content in the vessels of optic nerve, so the measurement of Hb levels can be useful for monitoring changes in optic nerve head (ONH), indirectly estimating its perfusion [15].

This editorial discusses the important findings from a recent prospective study [16]. In this research paper, the authors aimed to assess which of the two vascular systems mentioned above starts to be impaired in the initial phases of AD, thus representing an indicator for the early diagnosis of the disease [16]. In patients with mild AD, the authors investigated both vascular systems through the analysis of the choroidal thickness with OCT, the foveal avascular zone (FAZ) using OCT angiography (OCTA) and the Hb content in ONH by the Laguna ONhE program. As compared to controls, these patients exhibited a significant decrease in choroidal thickness in the subfoveal, temporal, nasal, superior and inferior points, suggesting that alterations in this ocular vascular layer are involved in the early AD pathogenesis [16]. It has been speculated that this thinner choroid may be related to a low perfusion and/or atrophic changes in this vascular network caused by the Aβ local deposition similar to what happens in the cerebral vascular system in AD patients [17]. As observed in the AD brains, the aggregation of Aβ peptides in the choroid may trigger an oxidative-induced inflammatory process that ultimately results in neurodegeneration and regression of choroidal vascularization [18,19,20]. Conversely, the authors did not find any differences in the FAZ and ONH Hb levels [16]. This can probably be explained by the fact that the vascular alterations occurring in AD pathogenesis may become visible in the retinal circulation only during the late stages of the disorder, as reported in patients with a more severe cognitive impairment [21].

Although this study lacks a longitudinal analysis monitoring the progression of vascular modifications, it provides important insights into the possibility of evaluating the choroidal thickness as an early biomarker for AD. A thinner choroid seems to be the main vascular change occurring in patients with mild AD while the retinal vascularization is not affected. Moreover, this study highlights the clinical application of ocular biomarkers for the prognosis and diagnosis of AD.

## Data Availability

Not applicable.
